# Oxyresveratrol: Structural Modification and Evaluation of Biological Activities

**DOI:** 10.3390/molecules21040489

**Published:** 2016-04-19

**Authors:** Nutputsorn Chatsumpun, Taksina Chuanasa, Boonchoo Sritularak, Vimolmas Lipipun, Vichien Jongbunprasert, Somsak Ruchirawat, Poonsakdi Ploypradith, Kittisak Likhitwitayawuid

**Affiliations:** 1 Department of Pharmacognosy and Pharmaceutical Botany, Faculty of Pharmaceutical Sciences, Chulalongkorn University, Bangkok 10330, Thailand; manee_many@hotmail.com (N.C.); s_taksina@yahoo.com (T.C.); Boonchoo.Sr@chula.ac.th (B.S.); Vichien.J@chula.ac.th (V.J.); 2 Department of Biochemistry and Microbiology, Faculty of Pharmaceutical Sciences, Chulalongkorn University, Bangkok 10330, Thailand; Vimolmas.L@chula.ac.th; 3 Laboratory of Medicinal Chemistry, Chulabhorn Research Institute, and Program in Chemical Biology, Chulabhorn Graduate Institute, 54 Kampaeng Phet 6 Road, Bangkok 10210, Thailand; somsak@cri.or.th

**Keywords:** oxyresveratrol, stilbene, free radicals, α-glucosidase, herpes simplex, cytotoxicity, *Artocarpus lacucha*, *Artocarpus lakoocha*

## Abstract

Oxyresveratrol (2,4,3′,5′-tetrahydroxystilbene, **1**), a phytoalexin present in large amounts in the heartwood of *Artocarpus lacucha* Buch.-Ham., has been reported to possess a wide variety of biological activities. As part of our continuing studies on the structural modification of oxyresveratrol, a library of twenty-six compounds was prepared via *O*-alkylation, aromatic halogenation, and electrophilic aromatic substitution. The two aromatic rings of the stilbene system of **1** can be chemically modulated by exploiting different protecting groups. Such a strategy allows for selective and exclusive modifications on either ring A or ring B. All compounds were evaluated *in vitro* for a panel of biological activities, including free radical scavenging activity, DNA protective properties, antiherpetic activity, inhibition of α-glucosidase and neuraminidase, and cytotoxicity against some cancer cell lines. Several derivatives were comparably active or even more potent than the parent oxyresveratrol and/or the appropriate positive controls. The partially etherified analogs 5′-hydroxy-2,3′,4-trimethoxystilbene and 3′,5′-dihydroxy-2,4-dimethoxystilbene demonstrated promising anti-herpetic and DNA protective activities, offering new leads for neuropreventive agent research, whereas 5′-hydroxy-2,3′,4,-triisopropoxystilbene displayed anti-α-glucosidase effects, providing a new lead molecule for anti-diabetic drug development. 3′,5′-Diacetoxy-2,4-diisopropoxystilbene showed potent and selective cytotoxicity against HeLa cancer cells, but the compound still needs further *in vivo* investigation to verify its anticancer potential.

## 1. Introduction

Stilbenes are a group of plant polyphenolic compounds derived from the mixed shikimate-acetate pathway. They are produced as phytoalexins, with limited but heterogeneous distribution in the plant kingdom [1]. Over the years, these C6-C2-C6 compounds, as well as their oligomers, have attracted significant research attention due to their elaborate and diverse structures, and wide-ranging biological activities [2]. Historically, these compounds have been studied for antioxidant, antibacterial, antiviral and anticancer activities [3,4]. However, recent efforts have been made to explore other potential uses such as therapeutic or preventive agents against non-communicable diseases (NCDs), for example, diabetes mellitus (DM), cardiovascular illnesses, and Parkinson’s disease (PD) [5,6,7].

**Scheme 1:**
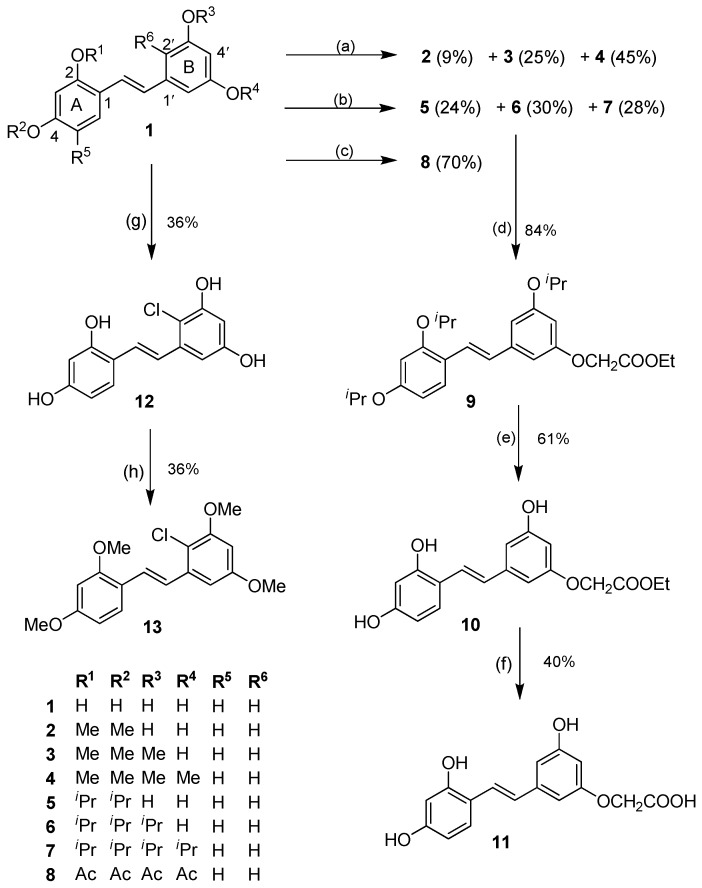
*O*-Alkylation, acylation, and aromatic chlorination of **1**. *Reagents and conditions*: (**a**) MeI, K_2_CO_3_, acetone, rt; (**b**) 2-bromopropane, K_2_CO_3_, DMF, 55 °C; (**c**) AcCl, Et_3_N, DMAP, CH_2_Cl_2_, rt; (**d**) ethyl bromoacetate, K_2_CO_3_, DMF, rt; (**e**) BCl_3_, CH_2_Cl_2_, −78 °C → rt, Ar; (**f**) 5% KOH, EtOH, rt; (**g**) NCS, AcOH (glacial), rt; (**h**) MeI, K_2_CO_3_, acetone, 55 °C.

Oxyresveratrol (2,4,3′,5′-tetrahydroxystilbene, **1**, Scheme 1) is present in large amounts in the heartwood of *Artocarpus lacucha* Buch.-Ham. (also known as *Artocarpus lakoocha* Roxb.) [8], and thus offers opportunities for chemical and biological studies. Our previous *in vitro* and *in vivo* experiments have shown that **1** is a potent tyrosinase inhibitor, and subsequent studies in human volunteers have confirmed it as an effective skin-whitening agent [9,10]. Despite its moderate *in vitro* inhibitory activity against herpes simplex virus type I (HSV-1) [11], compound **1**, in the form of topical cream, exhibited high therapeutic efficacy in mice cutaneously infected with HSV-1, with a potency comparable to that of the antiviral drug acyclovir [12]. Currently, mounting evidence has indicated HSV-1 infection in the brain as one of the key causative factors in the pathogenesis of Alzheimer’s disease (AD) [13], and this has suggested another medicinal importance for oxyresveratrol. Regarding the AD preventive potential, **1** has been reported to inhibit β-amyloid-induced neurotoxicity in cultured cortical neurons and *in vitro* activity of β-secretase, an enzyme vital for the production of the Aβ protein [14,15]. In addition, the compound has been studied for protective activity against Parkinson’s disease (PD) in cultured neuronal cells [16], and demonstrated to prevent neurotoxicity and DNA damage through its antioxidant properties [17,18]. Compound **1** showed modest inhibitory activity against H5N1 neuraminidase (an enzyme found in avian influenza virus) [19], but strong inhibition against α-glucosidase, an enzyme responsible for the breaking down of starch and polysaccharides into glucose in the intestine [20]. In spite of all the potential uses mentioned above, **1** seems to have rather limited clinical application. The compound has been reported to undergo extensive hepatic metabolism and rapid urinary elimination, resulting in a short half-life time, and thereby restricting its clinical use [21]. Moreover, its postulated function as a preventive agent for neurodegenerative disorders, such as AD or PD, appears to be hindered by its low ability to cross the blood-brain barrier (BBB) [22].

In our earlier report, we described a successful structural modification of **1**, which gave an analog with enhanced tyrosinase inhibitory activity [9]. In this investigation, we broadened our chemical studies and expanded the biological evaluations with the intention of improving the following biological activities of **1**: antioxidant and DNA protective properties, inhibitory activities against the enzymes α-glucosidase and neuraminidase, and ability to inhibit the growth of herpes simplex virus and some cancer cells.

## 2. Results and Discussion

### 2.1. Chemistry

#### 2.1.1. *O*-Alkylation/*O*-Acylation

When **1** was allowed to react with MeI in acetone at room temperature, di-, tri- and tetra-*O*-methylated products (**2**, **3** and **4**) were obtained in 9%, 25%, and 45% yields, respectively (Scheme 1). The alkylation of **1** with 2-bromopropane/K_2_CO_3_ in DMF gave the corresponding di-, tri- and tetra-*O*-isopropyl compounds (**5**, **6** and **7**) in 24%, 30%, and 28% yields, respectively (Scheme 1). It should be noted that it was difficult to avoid the formation of **7**, perhaps due to the greater solubility of the more alkylated ethers in DMF under the reaction conditions. In both *O*-alkylation reactions, the di-*O*-alkylation occurred on the two hydroxy groups of ring A, and tri-*O*-alkylation occupied both hydroxy groups of ring A and a hydroxy group of ring B. These results suggested that the phenolic groups on ring A were more reactive than their counterparts on ring B, most likely due to the conjugation of the hydroxy groups of ring A with the olefinic bond, which is not possible for those on ring B. This difference in the reactivity of the hydroxy groups was exploited in the subsequent studies on the aromatic electrophilic substitution reactions, in which the isopropyl group was found to be a better *O*-protecting group than the methyl after some experimentation (see below). Acetylation of **1** with AcCl gave **8** in 70% yield.

*O*-Alkylation of the tri-*O*-isopropyl compound **6** with ethyl bromoacetate under basic conditions went smoothly, to furnish in 84% yield the ethyl ester **9**, which then underwent BCl_3_-mediated deprotection of the isopropyl groups to give the trihydroxy compound **10** in 61% yield. Subsequent saponification of the ethyl ester using 5% KOH in ethanol provided the corresponding acid **11** in a moderate 40% yield (Scheme 1).

#### 2.1.2. Halogenation

It was expected that aromatic halogenation on **1** would be facile due to the high electron density contributed by the hydroxy groups. Due to the conjugation between the hydroxy groups on ring A and the olefin, the aromatic B ring should be more nucleophilic, particularly at the positions 2′/6′, and this difference should facilitate regioselective electrophilic aromatic halogenation under appropriate and mild conditions. Using *N*-chlorosuccinimide (NCS) and glacial acetic acid, the corresponding 2′-monochlorinated product **12** was obtained from **1** in 36% yield (Scheme 1). Chlorination on **7** and **20** (prepared from **5**) did not give the desired chlorinated product. Compound **12** smoothly underwent full methylation to give the tetramethylether **13** in 36% yield. Several attempts to carry out aromatic bromination on **1** or **4** with *N*-bromosuccinimide under various conditions were performed, but failed to give the brominated product. Iodination of **1** with *N*-iodosuccinimide was also not successful.

#### 2.1.3. Aromatic Electrophilic Substitution

**Scheme 2:**
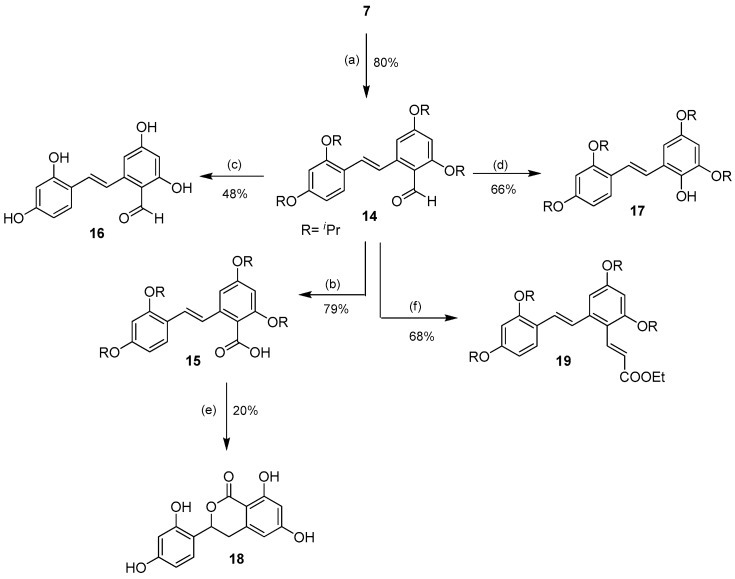
Selective aromatic formylation of ring B of **7** to **14** and the subsequent derivatizations of the aldehyde. *Reagents and conditions*: (**a**) POCl_3_, DMF, 0 °C, Ar; (**b**) NaClO_2_, NaH_2_PO_4_·2H_2_O, 2-methyl-2-butene, acetone, rt; (**c**) BCl_3_, CH_2_Cl_2_, −78 °C → rt, Ar; (**d**) *p*-TsOH·H_2_O, H_2_O_2_, 0 °C, Ar; (**e**) BBr_3_, CH_2_Cl_2_, −78 °C → rt, Ar; (**f**) EtOOCCH=PPh_3_, CH_2_Cl_2_, 0 °C.

When the tetra-*O*-isopropyl compound **7** reacted under the Vilsmeier-Haack formylation conditions, the corresponding ring *B*-formylated product **14** was obtained in good yield (80%) (Scheme 2).

The aldehyde functional group could be further oxidized to the corresponding carboxylic acid **15** in 79% yield using sodium chlorite. The aldehyde **14** smoothly underwent BCl_3_-mediated deprotection of the isopropyl groups to provide the 2′-formyloxyresveratrol **16** in moderate 48% yield. In addition, the aldehyde **14** could also undergo the acid-mediated Baeyer-Villiger-type Dakin reaction, which converted the aldehyde into the corresponding formate. Subsequent *in situ* cleavage of the formate under the reaction conditions furnished 2′-hydroxy-tetra-*O*-isopropyl oxyresveratrol **17** in good yield (66%). Unfortunately, all attempts to deprotect the isopropyl groups of **17** failed, and only a mixture of decomposed products was obtained. The deisopropylation of **15** with BBr_3_ under similar conditions unexpectedly gave the isochromanone **18** in 20% yield, a result of the Lewis acid-mediated removal of the *O*-isopropyl groups followed by C-O bond formation on the olefinic carbon of the stilbene system. In addition, the aldehyde **14** could undergo a smooth Wittig olefination to furnish the stilbenyl cinnamate **19** in 68% yield.

**Scheme 3:**
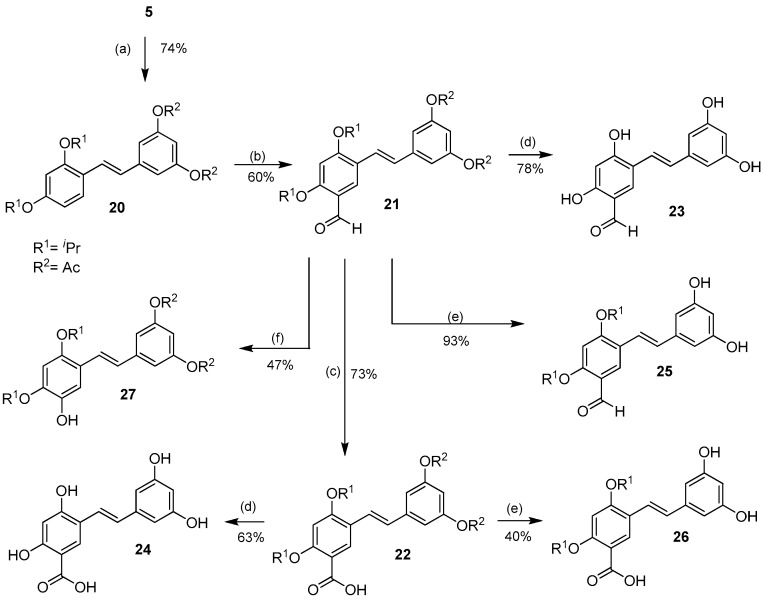
Protecting group-directed selective aromatic formylation of ring A of **20** to **21** and the subsequent derivatizations of the aldehyde. *Reagents and conditions*: (**a**) Ac_2_O, Et_3_N, CH_2_Cl_2_, rt; (**b**) POCl_3_, DMF, 0 °C, Ar; (**c**) NaClO_2_, NaH_2_PO_4_·2H_2_O, 2-methyl-2-butene, acetone, rt; (**d**) BCl_3_, CH_2_Cl_2_, −78 °C → rt, Ar; (**e**) 5% KOH, EtOH, rt; (**f**) *p*-TsOH·H_2_O, H_2_O_2_, 0 °C.

In order to effect the reactions on ring A of oxyresveratrol, the di-*O*-isopropyl compound **5** was acetylated under standard conditiond to provide ring A-di-*O*-isopropyl ring B-di-*O*-acetyl compound **20** in 74% yield (Scheme 3).

After some experimentation, ring A was formylated on the 5-position to give **21** exclusively, in 60% yield, under the best Vilsmeier-Haack conditions (10 eq of POCl_3_ at 0 °C → rt overnight under argon). Subsequent oxidation of the aldehyde **21** to the corresponding acid **22** using sodium chlorite gave the latter in 73% yield. BCl_3_-mediated cleavage of all the *O*-isopropyl groups as well as the *O*-acetyl groups of **21** and **22** finally gave the desired 5-formyl- and 5-carboxyoxyresveratrols **23** and **24** in 78% and 63% yields, respectively. Saponification of **21** and **22** using 5% KOH in ethanol to cleave the acetate groups gave 5-formyl- and 5-carboxy-di-*O*-isopropyl oxyresveratrol **25** and **26** in 93% and 40% yields, respectively. The aldehyde **21** was subjected to the Dakin reaction to give 5-hydroxy-di-*O*-acetyl-di-*O*-isopropyl oxyresveratrol **27** in 47% yield.

It is worth noting that the differences between protecting groups could be used to manipulate the selectivity of the aromatic electrophilic reactions. In the Vilsmeier-Haack reaction of the tetra-*O*-isopropyl compound **7**, all the protecting groups were electron-donating groups, which made the 2′ and 6′ positions of ring B the most reactive ones, but in the Vilsmeier-Haack reaction of the di-*O*-acetyl-di-*O*-isopropyl compound **20**, the acetyl groups were electron-withdrawing groups, and thus reduced the electron density of ring B. This was virtually equivalent to the deactivation of ring B, and therefore directed the formylation to occur on ring A. This synthetic strategy could be a useful tool for the synthesis of other similar analogs derivable from exploiting the difference in electron density of each aromatic ring.

### 2.2. Biological Activity

Since **1** was previously studied and reported to exhibit an array of biological activities including antioxidant activity and inhibition of the α-glucosidase and neuraminidase enzymes and HSV-1 (*vide supra*), all structurally modified analogs of **1** were subjected to the related assays to study the effects of such modification on each biological activity. In addition, these compounds were also evaluated for cytotoxic potential against four cancer cell lines of three different cancer types (breast, cervical and lung).

#### 2.2.1. Free Radical Scavenging Activity

First, examinations were conducted on the relationships between the phenolic and olefinic functionalities of the core stilbene structure and the free radical scavenging and DNA protective activities. Then, investigations were carried out to study how the nature and position of substituents with electron-withdrawing (*i.e.*, Cl, CHO and COOH) or electron-donating (*i.e.*, OH) properties on the aromatic rings affected these biological activities. Compounds were first evaluated at 100 μg/mL, and those that showed >80% inhibition were further analyzed to determine their IC_50_ values.

Oxyresveratrol (**1**) showed weaker DPPH free radical scavenging activity than Trolox (Table 1). The phenolic groups were essential since the activity was reduced or lost when some or all of the phenolic groups of **1** were esterified/etherified, as observed in compounds **2**–**8** and **20**. The only exception was compound **3**, the 2,4,3′-tri-*O*-methylated version of **1**, which showed higher activity against the superoxide anion radical than the parent compound. Interestingly, this compound also showed higher DNA protective properties.

The olefinic bridge, which provided conjugation (and thus electron delocalization) between the two aromatic rings, was also required for DPPH radical scavenging ability, as demonstrated from the marked decrease in activity of **18**. However, when evaluated against the superoxide anion, **18** displayed higher activity than **1**. This enhanced activity was most likely due to the electron-withdrawing effect of the C=O of the dihydropyrone ring which helped stabilize the negatively-charged stilbene radical formed from **18** after interaction with the superoxide anion.

Introducing an electron-donating group such as OH to the aromatic rings could restore the lost DPPH scavenging activity caused by phenolic esterification/etherification. This was inferred by comparing the activity of the following pairs of structures: **7** (no activity) *vs.* **17**, and **20** (no activity) *vs.* **27**. However, these sets of compounds did not give similar results in the superoxide assay.

Placing an electron-withdrawing substituent, such as Cl, CHO and COOH, onto the aromatic ring of **1** yielded compounds **12**, **16**, **23** and **24** which showed weaker activity against the DPPH radical, but enhanced activity against the superoxide anion. The improved activity was likely attributable to the electron-withdrawing nature of these groups that helped to distribute the negative charge of the radicals generated from the reactions of these stilbenoids with the superoxide anion.

#### 2.2.2. DNA Protective Property

Riboflavin (RF), when exposed to light, generates several kinds of free radicals, including triplet (^3^RF•), neutral oxidized (RF(−H)•) and cation (RF•+) riboflavin radical. The (^3^RF•) radical then transfers energy to oxygen to form reactive oxygen species (ROS) such as singlet oxygen (^1^O_2_) and superoxide (O_2_•−) radicals [23,24]. These free radicals, with their high energy, can each damage DNA by attacking the purine base guanine to form 8-hydroxyguanine and 2,6-diamino-4-hydroxy-5-formamidopyrimidine [23,25,26,27]. Any compound that can efficiently protect DNA from the damage induced by photosensitized riboflavin must be capable of scavenging or neutralizing these free radicals. In this study, compounds were first evaluated at 100 μg/mL, and those with >80% inhibition were further analyzed for their IC_50_ values (Table 1).

Compound **1** showed higher DNA protective activity than Trolox, despite its inferior activity in the DPPH assay (Table 1). Total *O*-alkylation or *O*-acylation of **1** destroyed the activity, as observed in the lost or diminished activity for **4**, **7**, **8**, **9**, **14**, **15**, **17**, **19** and **20**. The lost activity of **20** could be rescued by placing an OH substituent on ring B (**27**), an outcome similar to the case of the DPPH radical. The partially *O*-alkylated products of **1**, including **3**, **10**, and **11** showed enhanced DNA protective activity, but the tri-*O*-isopropylated product **6** displayed markedly reduced activity.

Compound **18** did not lose the DNA protective activity despite the removal of the C=C bond. With the aid of the C=O of the dihydro-α-pyrone ring, this compound showed even higher activity than **1**. The placement of the electron-donating group OH on ring A of **20** could restore the lost activity, giving **27**, which is a better DNA protectant than **1**. The introduction of an electron-withdrawing substituent to the aromatic rings of **1** gave unclear results. Placing Cl on ring B (*i.e*., compound **12**) slightly increased the activity, but introducing COOH onto ring A reduced the activity (compound **24**).

#### 2.2.3. α-Glucosidase Inhibitory Activity

Almost all of the stilbenoids **2**−**27** prepared from oxyresveratrol exhibited reduced or lost inhibitory activity against α-glucosidase (the positive control acarbose: IC_50_ 745.9 ± 88.4 μM). The only exception was **6**, which showed activity (IC_50_ 19.6 ± 3.6 μM) comparable to that of **1** (IC_50_ 19.5 ± 1.7 μM). In a previous study, **1** has been suggested to be a promising structure for anti-diabetic drug development [7]. However, the compound, after oral absorption, was found to undergo extensive hepatic metabolism and rapid urinary elimination, resulting in a short half-life time (~0.96 h) [21]. Compound **6**, with three isopropoxy groups, is predicted to have much slower metabolism and may be considered as a better candidate for the preclinical and clinical studies for anti-DM drugs.

#### 2.2.4. Neuraminidase Inhibitory Activity

In this study, oxyresveratol and all of its derivatives **2**–**27** exhibited no activity against the enzyme neuraminidase (positive control oseltamivir, IC_50_ 0.5 nM), although **1** has been earlier described as having modest activity [19]. It should be mentioned that resveratrol, a closely related stilbene, and several of its synthetic and semisynthetic analogs have been recently reported to possess significant inhibitory activity against influenza neuraminidase [28].

#### 2.2.5. Antiherpetic Activity

Compounds **1**–**27** were first evaluated for anti-HSV-1 activity at 100 μg/mL. Those that showed >50% inhibition at this concentration were further studied to determine their IC_50_ values (Table 2).

Three analogs, including compounds **2**, **3**, and **6**, showed IC_50_ values significantly lower than that of **1**. Stilbenes **2** and **3** (di- and tri-*O*-methylated products of **1**) displayed 3- to 4-fold higher antiherpetic activity than the parent compound. Recently, compelling evidence has suggested that HSV-1 infection of neuronal and glial cells plays a pivotal role in the pathogenesis of Alzheimer’s disease (AD), and the use of antiviral drugs in the treatment of AD has been proposed [13]. As mentioned earlier, **1**, despite its moderate anti-herpetic activity *in vitro*, exhibited potent activity *in vivo* in mice cutaneously infected with HSV-1 [12].However, due to its poor BBB permeability [22], oxyresveratrol (**1**) seems to have limited clinical application. In this regard, stilbenes **2** and **3**, with their higher lipophilicity, may be better candidates for further investigation on HSV infection of the CNS, as well as neurodegenerative diseases such as AD or PD.

#### 2.2.6. Cytotoxicity against Cancer Cells

All compounds (**1**–**27**) were initially evaluated at 50 μg/mL, and those demonstrating >50% inhibition were subjected to IC_50_ determination (Table 3).

Seventeen derivatives demonstrated higher cytotoxicity than the parent compound **1**, which was virtually non-cytotoxic against all cancer cell lines. The partially (di- or tri-) *O*-alkylated products (**2**, **3**, **5** and **6**) showed increased cytotoxicity against all types of cancer cells. The fully *O*-methylated product **4** showed enhanced selective cytotoxicity against HeLa and H69AR cells, but no activity against T47-D and A549 cells. Interestingly, the IC_50_ value of **4** against the multidrug-resistant small lung cancer cells (H69AR) was in the same range as that of doxorubicin (*p* > 0.05). The fully *O*-isopropylated products, including **7**, **9**, **17**, and **19**, did not show cytotoxicity in the assays, probably a result of their poor aqueous solubility. With regard to the esterified analogs, the fully *O*-acetylated compound **8** showed slightly improved activity selectively against T47-D cells, but less activity against HeLa and A549 cells when compared with **1**. Compound **20**, a di-*O*-isopropylated-di-*O*-acetylated product of **1**, showed better cytotoxicity than **1** in all cancer cell lines, with selective potency against HeLa cells.

Different impacts on the cytotoxicity were observed when an electron-withdrawing substituent was attached to the aromatic rings of **1**. Chlorination of **1** at C-2′ gave **12**, a product with increased cytotoxicity against almost all cancer cell lines, except T47D cells. Similar cytotoxicity profiles were observed for compounds **16** and **23**, the 5- and the 2′-formylated analogs, both of which displayed increased activity against T47-D, HeLa and A549, but no activity against H69AR cells. Further structural modification of **23** by converting the CHO into a COOH group (compound **24**) destroyed the cytotoxicity in all cancer cell lines. When **7**, the fully *O*-isopropylated analog, was formylated or carboxylated at C-2′, partial restoration of cytotoxicity was observed (compounds **14** and **15**). For **20**, placing a CHO group at C-5 gave a product **21** with mixed results since the cytotoxicity against TD47-D cells was enhanced, but the activity against HeLa A549 and H69AR cells was weakened (*p* < 0.05). Transforming the CHO at this position of **21** into a COOH group abolished the activity (compound **22**). An electron-donating group such as hydroxy group, when added to the aromatic rings, could not induce cytotoxicity, as reflected from the non-cytotoxicity of both **7** and **17**, but instead seemed to weaken the cytotoxicity, as evident when comparing the strong activity of **20** with that of **27** which showed less cytotoxicity in HeLa and A549 cancer cells (*p* < 0.05).

## 3. Materials and Methods

### 3.1. General Information 

All chemical reagents were purchased from commercial suppliers. Melting points (uncorrected) were determined on a Fisher-Johns hot stage melting point apparatus. IR spectra were performed using Universal Attenuated Total Reflectance (UATR) on a 2000 FT-IR system (Perkin Elmer, Boston, MA, USA) or an A-30 spectrophotometer (Jasco, Easton, MD, USA). High resolution mass spectra were taken on a MicroTOF instrument (Bruker, Billerica, MA, USA) using atmospheric pressure chemical ionization (APCI) or electrospray ionization (ESI) in positive or negative mode. ^1^H- and ^13^C-NMR were recorded on an Avance III 300 MHz spectrometer or 400 MHz spectrometer (Bruker, Billerica, MA, USA). All chemical shift values were reported as δ (ppm) and coupling constant values *J* were measured in Hz. Oxyresveratrol (1) was isolated and purified from the heartwood of *Artocarpus lacucha* Buch.-Ham. (*A. lakoocha* Roxb.) as previously described [11,29,30].

### 3.2. Synthesis

#### 3.2.1. Preparation of Compounds **2**–**4**

K_2_CO_3_ (680 mg, 4.92 mmol) and MeI (0.11 mL, 1.8 mmol) were added to the solution of **1** (200 mg, 0.82 mmol) in acetone (4 mL) at room temperature. The reaction mixture was stirred overnight. Then, the reaction mixture was diluted with water (5 mL) and extracted with EtOAc (3 × 5 mL). The EtOAc layer was washed with brine, dried over anhydrous Na_2_SO_4_, filtered and concentrated under reduced pressure to give a crude product which, after purification by preparative TLC (50% EtOAc/hexanes), furnished **2** (19 mg, 9%), **3** (60 mg, 25%) and **4** (112 mg, 45%).

*3′,5′-Dihydroxy-2,4-dimethoxystilbene* (**2**): viscous brown oil; IR (UATR, cm^−1^): 3374, 2931, 1601; ^1^H-NMR (300 MHz, CDCl_3_), δ (ppm): 7.43 (d, *J* = 8.4 Hz, 1H), 7.30 (d, *J* = 16.5 Hz, 1H), 6.82 (d, *J* = 16.2 Hz, 1H), 6.55 (d, *J* = 2.1 Hz, 2H), 6.48 (dd, *J* = 8.7, 2.1 Hz, 1H), 6.44 (d, *J* = 2.1 Hz, 1H), 6.24 (t, *J* = 2.1 Hz, 1H), 3.82 (s, 3H), 3.80 (s, 3H); ^13^C-NMR (75 MHz, CDCl_3_), δ (ppm): 160.6, 158.1, 156.9, 141.0, 127.3, 126.2, 124.1, 119.2, 105.9, 105.1, 101.7, 98.5, 55.5, 55.4; TOF-HRMS *m/z* [M + H]^+^, calcd for C_16_H_17_O_4_: 273.1121; found: 273.1118.

*5′-Hydroxy-2,3′,4-trimethoxystilbene* (**3**): viscous brown oil; IR (UATR, cm^−1^): 3408, 2938, 1590; ^1^H-NMR (300 MHz, CDCl_3_), δ (ppm): 7.46 (d, *J* = 8.4 Hz, 1H), 7.33 (d, *J* = 16.5 Hz, 1H), 6.88 (d, *J* = 16.5 Hz, 1H), 6.62 (d, *J* = 1.8 Hz, 1H), 6.60 (d, *J* = 1.8 Hz, 1H), 6.49 (dd, *J* = 8.4, 2.4 Hz, 1H), 6.45 (d, *J* = 2.4 Hz, 1H), 6.30 (t, *J* = 2.1 Hz, 1H), 3.83 (s, 3H), 3.81 (s, 3H), 3.78 (s, 3H); ^13^C-NMR (75 MHz, CDCl_3_), δ (ppm): 160.9, 160.5, 158.0, 156.9, 140.6, 127.3, 126.6, 123.9, 119.3, 105.8, 105.0, 104.6, 100.4, 98.5, 55.5, 55.4, 55.3; TOF-HRMS *m/z* [M + H]^+^, calcd for C_17_H_19_O_4_: 287.1278; found: 287.1285.

*2,3′,4,5′-Tetramethoxystilbene* (**4**): white solid, mp 65–68 °C; ^1^H-NMR (300 MHz, CDCl_3_), δ (ppm): 7.46 (d, *J* = 8.4 Hz, 1H), 7.36 (d, *J* = 16.2 Hz, 1H), 6.92 (d, *J* = 16.5 Hz, 1H), 6.66 (d, *J* = 2.4 Hz, 2H), 6.47 (dd, *J* = 8.4, 2.4 Hz, 1H), 6.42 (d, *J* = 2.4 Hz, 1H), 6.34 (t, *J* = 2.1 Hz, 1H), 3.81 (s, 3H), 3.79 (s, 6H), 3.77 (s, 3H); ^13^C-NMR (75 MHz, CDCl_3_), δ (ppm): 160.8, 160.5, 158.0, 140.3, 127.2, 126.8, 123.7, 119.1, 104.9, 104.2, 99.2, 98.3, 55.3, 55.2, 55.1; TOF-HRMS *m/z* [M + H]^+^, calcd for C_18_H_21_O_4_: 301.1434; found: 301.1440. The spectroscopic data were in agreement with previously reported values [9].

#### 3.2.2. Preparation of Compounds **5**–**7**

To a solution of **1** (100 mg, 0.41 mmol) in DMF (2 mL), K_2_CO_3_ (283 mg, 2.05 mmol) and 2-bromopropane (2.30 mL, 24.6 mmol) were added. Then 2-bromopropane (1.15 mL, 12.3 mmol) was added every 24 h. The reaction mixture was stirred at 55 °C for 3 days and monitored by TLC. After completion, water (5 mL) was added, and the reaction was extracted with EtOAc (3 × 5 mL). The organic phase was washed with water (7 × 5 mL) and brine, dried over Na_2_SO_4_, filtered and concentrated. Column chromatographic purification with gradient EtOAc/hexanes (10% EtOAc/hexanes → 80% EtOAc/hexanes) gave **5** (32 mg, 24%), **6** (46 mg, 30%) and **7** (47 mg, 28%).

*3′,5′-Dihydroxy-2,4-diisopropoxystilbene* (**5**): brown oil; IR (UATR, cm^−1^): 3379, 2976, 1598; ^1^H-NMR (400 MHz, CDCl_3_), δ (ppm): 7.43 (d, *J* = 8.4 Hz, 1H), 7.31 (d, *J* = 16.4 Hz, 1H), 6.84 (d, *J* = 16.4 Hz, 1H), 6.56 (d, *J* = 2.1 Hz, 2H), 6.48 (dd, *J* = 8.5, 2.3 Hz, 1H), 6.45 (d, *J* = 2.2 Hz, 1H), 6.25 (br s, 1H), 4.52 (m, 2H), 1.35 (d, *J* = 6 Hz, 6H), 1.33 (d, *J* = 6 Hz, 6H); ^13^C-NMR (100 MHz, CDCl_3_), δ (ppm): 158.6, 157.0, 156.5, 141.0, 127.4, 126.1, 124.3, 120.3, 107.5, 105.8, 103.2, 101.7, 71.2, 70.1, 22.1, 22.0; TOF-HRMS *m/z* [M − H]^−^, calcd for C_20_H_23_O_4_: 327.1602; found: 327.1594.

*5′-Hydroxy-2,3′,4,-triisopropoxystilbene* (**6**): brown oil; IR (UATR, cm^−1^): 3393, 2976, 1588; ^1^H-NMR (400 MHz, CDCl_3_), δ (ppm): 7.45 (d, *J* = 8.5 Hz, 1H), 7.32 (d, *J* = 16.4 Hz, 1H), 6.88 (d, *J* = 16.4 Hz, 1H), 6.61 (t, *J* = 1.5 Hz, 1H), 6.56 (t, *J* = 1.6 Hz, 1H), 6.49 (dd, *J* = 8.5, 2.3 Hz, 1H), 6.46 (d, *J* = 2.3 Hz, 1H), 6.28 (t, *J* = 2.1 Hz, 1H), 4.534 (m, 2H), 4.527 (m, 1H), 1.37 (d, *J* = 6 Hz, 6H), 1.343 (d, *J* = 6 Hz, 6H), 1.336 (d, *J* = 6 Hz, 6H); ^13^C-NMR (100 MHz, CDCl_3_), δ (ppm): 159.2, 158.6, 156.7, 156.5, 140.8, 127.4, 126.2, 124.4, 120.2, 107.4, 107.0, 105.4, 103.0, 101.8, 71.0, 70.0, 69.9, 22.2, 22.0; TOF-HRMS *m/z* [M − H]^−^, calcd for C_23_H_29_O_4_: 369.2071; found: 369.2066.

*2,3′,4,5′-Tetraisopropoxystilbene* (**7**): white solid, mp 58–60 °C; IR (UATR, cm^−1^): 2976, 1585; ^1^H-NMR (300 MHz, CDCl_3_), δ (ppm): 7.46 (d, *J* = 8.4 Hz, 1H), 7.33 (d, *J* = 16.4 Hz, 1H), 6.91 (d, *J* = 16.4 Hz, 1H), 6.62 (d, *J* = 2.2 Hz, 2H), 6.49 (dd, *J* = 8.5, 2.3 Hz, 1H), 6.45 (d, *J* = 3.9 Hz, 1H), 6.34 (t, *J* = 2.1 Hz, 1H), 4.55 (m, 4H), 1.37 (d, *J* = 6 Hz, 6H), 1.35 (d, *J* = 6 Hz, 12H), 1.34 (d, *J* = 6 Hz, 6H); ^13^C-NMR (75 MHz, CDCl_3_), δ (ppm): 159.1, 158.6, 156.6, 140.5, 127.4, 126.7, 124.2, 120.4, 107.3, 106.4, 103.0, 102.5, 71.0, 69.9, 69.8, 22.2, 22.1; TOF-HRMS *m/z* [M + H]^+^, calcd for C_26_H_37_O_4_: 413.2686; found: 413.2679.

#### 3.2.3. Preparation of 2,3′,4,5′-Tetraacetoxystilbene (**8**)

Compound **8** was prepared as previously described [31]. To a well-stirred solution of **1** (50 mg, 0.21 mmol) in CH_2_Cl_2_ (2 mL), Et_3_N (0.17 mL, 1.23 mmol), DMAP (63 mg, 0.51 mmol) and acetyl chloride (0.04 mL, 0.61 mmol) were added at room temperature, and the reaction mixture was stirred for 3 h. After completion of the reaction, it was extracted with EtOAc (3 × 5 mL). The EtOAc layer was washed with brine, dried over Na_2_SO_4_, filtered and concentrated under reduced pressure to give a crude product. Purification with column chromatography eluting with 40% EtOAc/hexanes gave **8** (60 mg, 70%). White solid, mp 129–131 °C; IR (UATR, cm^−1^): 1762, 1605; ^1^H-NMR (300 MHz, CDCl_3_), δ (ppm): 7.61 (d, *J* = 8.7 Hz, 1H), 7.08 (d, *J* = 2.1 Hz, 2H), 7.05 (d, *J* = 15.9 Hz, 1H), 7.03 (dd, *J* = 8.4, 2.4 Hz, 1H), 6.97 (d, *J* = 15.9 Hz, 1H), 6.95 (d, *J* = 2.1 Hz, 1H), 6.85 (t, *J* = 2.1 Hz, 1H), 2.36 (s, 3H), 2.31 (s, 6H), 2.29 (s, 3H); ^13^C-NMR (75 MHz, CDCl_3_), δ (ppm): 168.93, 168.89, 168.8, 151.3, 150.5, 148.5, 139.4, 129.5, 127.2, 127.1, 123.5, 119.5, 117.0, 116.4, 114.8, 21.10, 21.09, 20.99; TOF-HRMS *m/z* [M + Na]^+^, calcd for C_22_H_20_NaO_8_: 435.1056; found: 435.1050. The spectroscopic data were in agreement with earlier reported values [32].

#### 3.2.4. Preparation of 3′-*O*-Carbethoxymethyl-2,4,5′-triisopropoxystilbene (**9**)

To a solution of **6** (100 mg, 0.27 mmol) in DMF (4 mL), K_2_CO_3_ (56 mg, 0.40 mmol) and ethyl bromoacetate (0.04 mL, 0.4 mmol) were added at room temperature, and the reaction mixture was stirred overnight. After completion of the reaction, water (5 mL) was added and the mixture was extracted with EtOAc (3 × 5 mL). The organic layer was washed with water (7 × 5 mL) and brine, dried over Na_2_SO_4_, filtered and concentrated under reduced pressure to give a crude product, which was purified by column chromatography on silica (15% EtOAc/hexanes) to furnish **9** (103 mg, 84%). Light brown oil; IR (UATR, cm^−1^): 2977, 1760, 1588; ^1^H-NMR (300 MHz, CDCl_3_), δ (ppm): 7.45 (d, *J* = 8.4 Hz, 1H), 7.33 (d, *J* = 16.5 Hz, 1H), 6.90 (d, *J* = 16.5 Hz, 1H), 6.68 (d, *J* = 1.5 Hz, 1H), 6.62 (d, *J* = 1.8 Hz, 1H), 6.48 (dd, *J* = 8.7, 2.4 Hz, 1H), 6.45 (d, *J* = 2.1 Hz, 1H), 6.36 (t, *J* = 2.2 Hz, 1H), 4.62 (s, 2H), 4.54 (m, 3H), 4.26 (q, *J* = 7.1 Hz, 2H), 1.38 (d, *J* = 6 Hz, 6H), 1.344 (d, *J* = 6 Hz, 6H), 1.339 (d, *J* = 6 Hz, 6H), 1.30 (t, *J* = 6.8 Hz, 3H); ^13^C-NMR (75 MHz, CDCl_3_), δ (ppm): 168.9, 159.0, 158.7, 156.6, 140.7, 127.4, 126.3, 124.6, 120.1, 107.6, 107.3, 104.7, 102.9, 101.4, 70.9, 69.9, 65.5, 61.8, 61.3, 22.1, 22.0, 14.1; TOF-HRMS *m/z* [M + Na]^+^, calcd for C_27_H_36_NaO_6_: 479.2404; found: 479.2388.

#### 3.2.5. Preparation of 3′-*O*-Carbethoxymethyl-2,4,5′-trihydroxystilbene (**10**)

A solution of BCl_3_ (3.07 mL, 3. 07 mmol) was added to a solution of **9** (233 mg, 0.51 mmol) in CH_2_Cl_2_ (8 mL) at −78 °C under argon. Then it was allowed to warm to room temperature and stirred overnight. The reaction mixture was quenched with water (10 mL) and then extracted with EtOAc (3 × 10 mL). The organic phase was washed with brine, dried over Na_2_SO_4_, filtered and concentrated under reduced pressure to give a crude product which was purified by preparative TLC (50% EtOAc/hexanes) to give **10** (102 mg, 61%). Pale yellow solid, mp 198–200 °C; IR (UATR, cm^−1^): 3369, 1731, 1591; ^1^H-NMR (300 MHz, acetone-*d*_6_), δ (ppm): 7.43 (d, *J* = 8.4 Hz, 1H), 7.36 (d, *J* = 16.5 Hz, 1H), 6.97 (d, *J* = 16.5 Hz, 1H), 6.70 (br s,1H), 6.63 (br s,1H), 6.49 (d, *J* = 2.1 Hz, 1H), 6.49 (br s, 1H), 6.43 (dd, *J* = 8.4, 2.1 Hz, 1H), 6.34 (t, *J* = 2.1 Hz, 1H), 4.70 (s, 2H), 4.21 (q, *J* = 6.9 Hz, 2H), 1.25 (t, *J* = 6.9 Hz, 3H); ^13^C-NMR (75 MHz, acetone-*d*_6_), δ (ppm): 169.4, 160.4, 159.4, 159.1, 156.9, 141.7, 128.4, 126.0, 124.9, 117.1, 108.4, 107.1, 104.4, 103.5, 101.6, 65.8, 61.3, 14.4; TOF-HRMS *m/z* [M + Na]^+^, calcd for C_18_H_18_NaO_6_: 353.0996; found: 353.0989.

#### 3.2.6. Preparation of 3′-*O*-Carboxymethyl-2,4,5′-trihydroxystilbene (**11**)

To **10** (13 mg, 0.03 mmol), KOH (5% in EtOH, 1 mL) was added at room temperature, and the reaction mixture was stirred for 10 min. The reaction was acidified with 2 N HCl to pH 5. The material was extracted with EtOAc (3 × 5 mL), and the combined organic layers were dried over Na_2_SO_4_, filtered, and concentrated under reduced pressure to provide **11** (3 mg, 40%). Brown solid; IR (UATR, cm^−1^): 3322, 2921, 2851, 1713; ^1^H-NMR (300 MHz, methanol-*d*_4_), δ (ppm): 7.33 (d, *J* = 8.9 Hz, 1H), 7.30 (d, *J* = 16.4 Hz, 1H), 6.86 (d, *J* = 16.4 Hz, 1H), 6.58 (d, *J* = 1.6 Hz, 1H), 6.56 (d, *J* = 1.8 Hz, 1H), 6.31 (dd, *J* = 5.9, 2.3 Hz, 1H), 6.30 (s, 1H), 6.25 (t, *J* = 2.1 Hz, 1H), 4.63(s, 2H); ^13^C-NMR (75 MHz, methanol-*d*_4_), δ (ppm):172.9, 160.8, 159.7, 159.4, 157.4, 142.4, 128.5, 126.2, 125.4, 117.7, 108.4, 107.3, 104.8, 103.6, 101.7, 65.9; TOF-HRMS *m/z* [M − H]^−^, calcd for C_16_H_13_O_6_: 301.0718; found: 301.0708.

#### 3.2.7. Preparation of 2′-Chloro-2,3′,4,5′-tetrahydroxystilbene (**12**)

A mixture of **1** (100 mg, 0.41 mmol) and *N*-chlorosuccinimide (54.8 mg, 0.41 mmol) in glacial acetic acid (4 mL) was stirred at room temperature under argon for 3 h. After completion, solvent was removed under reduced pressure. Purification by column chromatography (10% MeOH/CH_2_Cl_2_) gave of **12** (42 mg, 36% yield). Light brown solid, mp 185–187 °C; IR (UATR, cm^−1^): 3196, 1599; ^1^H-NMR (300 MHz, acetone-*d*_6_), δ (ppm): 7.44 (d, *J* = 8.4 Hz, 1H), 7.38 (d, *J* = 16.4 Hz, 1H), 7.32 (d, *J* = 16.4 Hz, 1H), 6.78 (d, *J* = 2.7 Hz, 1H), 6.45 (d, *J* = 2.4 Hz, 1H), 6.43 (d, *J* = 2.7 Hz, 1H), 6.41 (dd, *J* = 8.4, 2.4 Hz, 1H); ^13^C-NMR (75 MHz, acetone-*d*_6_), δ (ppm): 158.6, 156.5, 156.3, 153.9, 137.9, 127.9, 126.3, 121.3, 116.2, 110.4, 107.6, 103.8, 102.7, 102.3; TOF-HRMS *m/z* [M + H]^+^, calcd for C_14_H_12_ClO_4_: 279.0419; found: 279.0408.

#### 3.2.8. Preparation of 2′-Chloro-2,3′,4,5′-tetramethoxystilbene (**13**)

To a well-stirred solution of **12** (88 mg, 0.3 mmol) in acetone (15 mL), K_2_CO_3_ (265 mg, 1.92 mmol) and MeI (0.11 mL, 1.8 mmol) were added. The reaction mixture was stirred at 55 °C overnight and monitored by TLC. After completion of the reaction, the mixture was diluted with water (10 mL), and extracted with EtOAc (3 × 10 mL). The organic phase was washed with brine. The organic extract was dried over Na_2_SO_4_, filtered and evaporated *in vacuo*. The residue obtained was purified over silica gel using 20% EtOAc/hexanes to furnish **13** (40 mg, 36%). White solid; ^1^H-NMR (300 MHz, CDCl_3_), δ (ppm): 7.56 (d, *J* = 8.4 Hz, 1H), 7.44 (d, *J* = 16.5 Hz, 1H), 7.33 (d, *J* = 16.5 Hz, 1H), 6.83 (d, *J* = 2.7 Hz, 1H), 6.52 (dd, *J* = 8.7, 2.1 Hz, 1H), 6.46 (d, *J* = 2.1 Hz, 1H), 6.42 (t, *J* = 2.7 Hz, 1H), 3.87 (s, 3H), 3.86 (s, 3H), 3.85 (s, 3H), 3.83 (s, 3H); ^13^C-NMR (75 MHz, CDCl_3_), δ (ppm): 160.9, 158.6, 158.2, 155.9, 137.7, 127.7, 126.0, 123.2, 119.2, 113.8, 105.0, 101.9, 98.6, 98.4, 56.2, 55.6, 55.5, 55.4; TOF-HRMS *m/z* [M]^+^, calcd for C_18_H_20_ClO_4_: 335.1045; found: 335.1035.

#### 3.2.9. Preparation of 2′-Formyl-2,3′,4,5′-tetraisopropoxystilbene (**14**)

POCl_3_ (0.16 mL, 1.69 mmol) was stirred with dry DMF (4 mL) at room temperature for 2 h under argon. A solution of **7** (200 mg, 0.48 mmol) in dry DMF (4 mL) was added at 0 °C and the reaction mixture was further stirred overnight. After completion of the reaction, cool water (10 mL) was added, and the mixture was extracted with EtOAc (3 × 10 mL). The organic layer was washed with water (7 × 10 mL) and brine, dried over Na_2_SO_4_, filtered and concentrated under reduced pressure to give a crude product, which was purified by column chromatography on silica (15% EtOAc/hexanes) to furnish **14** (171 mg, 80%). Viscous yellow oil; IR (UATR, cm^−1^): 2976, 1671, 1586; ^1^H-NMR (300 MHz, CDCl_3_), δ (ppm): 10.53 (s, 1H), 8.08 (d, *J* = 16.4 Hz, 1H), 7.60 (d, *J* = 8.6 Hz, 1H), 7.30 (d, *J* = 16.4 Hz, 1H), 6.75 (d, *J* = 2.1 Hz, 1H), 6.50 (dd, *J* = 8.6, 2.3 Hz, 1H), 6.44 (d, *J* = 2.4 Hz, 1H), 6.35 (d, *J* = 2.1 Hz, 1H), 4.69 (sept, *J* = 6 Hz, 1H), 4.48–4.63 (m, 3H), 1.33–1.41 (m, 24H); ^13^C-NMR (75 MHz, CDCl_3_), δ (ppm): 191.1, 163.6, 162.7, 158.9, 156.7, 143.9, 128.1, 127.3, 125.4, 120.4, 116.8, 107.4, 105.0, 102.9, 100.1, 71.4, 70.9, 70.1, 69.9, 22.2, 22.04, 21.98; TOF-HRMS *m/z* [M + H]^+^, calcd for C_27_H_37_O_5_: 441.2635; found: 441.2639.

#### 3.2.10. Preparation of 2′-Carboxy-2,3′,4,5′-tetraisopropoxystilbene (**15**)

A solution of NaClO_2_ (260 mg, 2.88 mmol) and NaH_2_PO_4_·2H_2_O (447 mg, 2.86 mmol) in water (1.5 mL) was added to the solution of **14** (157 mg, 0.36 mmol) and 2-methyl-2-butene (0.13 mL, 1.5 mmol) in acetone (1.5 mL) at room temperature. The reaction mixture was stirred for 1 h. After completion, water (5 mL) was added, and the reaction mixture was extracted with EtOAc (3 × 5 mL). The EtOAc phase was washed with brine, dried over Na_2_SO_4_, filtered and concentrated under reduced pressure to give crude product, which was purified by column chromatography on silica (40% EtOAc/hexanes) to furnish **15** (129 mg, 79%). Pale yellow solid, mp 154–156 °C; IR (UATR, cm^−1^): 2976, 1730, 1697, 1592; ^1^H-NMR (300 MHz, CDCl_3_), δ (ppm): 7.85 (d, *J* = 16.2 Hz, 1H), 7.56 (d, *J* = 8.4 Hz, 1H), 7.19 (d, *J* = 16.2 Hz, 1H), 6.85 (d, *J* = 2.1 Hz, 1H), 6.49 (dd, *J* = 8.4, 2.1 Hz, 1H), 6.44 (d, *J* = 2.1 Hz, 1H), 6.42 (d, *J* = 2.1 Hz, 1H), 4.68 (m, 2H), 4.54 (m, 2H), 1.44 (d, *J* = 6.3 Hz, 6H), 1.40 (d, *J* = 6.3 Hz, 6H), 1.37 (d, *J* = 6.3 Hz, 6H), 1.34 (d, *J* = 6 Hz, 6H); ^13^C-NMR (75 MHz, CDCl_3_), δ (ppm): 166.0, 160.9, 158.8, 158.0, 156.6, 145.6, 128.1, 126.6, 126.5, 120.4, 107.4, 103.0, 101.5, 74.1, 71.0, 70.2, 70.0, 22.2, 22.1, 22.0, 21.9; TOF-HRMS *m/z* [M + H]^+^, calcd for C_27_H_37_O_6_: 457.2585; found: 457.2587.

#### 3.2.11. Preparation of 2′-Formyl-2,3′,4,5′-tetrahydroxystilbene (**16**)

A solution of BCl_3_ (2.17 mL, 2.17 mmol) was added to a solution of **14** (119 mg, 0.27 mmol) in CH_2_Cl_2_ (4 mL) at −78 °C under argon. The reaction was allowed to warm up to room temperature and stirred overnight. Then, water (5 mL) was added, and the reaction was extracted with EtOAc (3 × 5 mL). The EtOAc layer was washed with brine, dried over Na_2_SO_4_, filtered and concentrated under reduced pressure to give a crude product which was purified by using preparative TLC (40% EtOAc/hexanes) to give **16** (35 mg, 48%). Yellow solid, decomposed >218 °C; IR (UATR, cm^−1^): 3337, 1602; ^1^H-NMR (300 MHz, methanol-*d*_4_), δ (ppm): 10.17 (s, 1H), 7.55 (d, *J* = 15.9 Hz, 1H), 7.38 (d, *J* = 9 Hz, 1H), 7.23 (d, *J* = 15.9 Hz, 1H), 6.56 (d, *J* = 2 Hz, 1H), 6.33 (dd, *J* = 8.6, 2.3 Hz, 1H), 6.32 (br s, 1H), 6.14 (d, *J* = 2 Hz, 1H); ^13^C-NMR (75 MHz, methanol-*d*_4_), δ (ppm): 194.3, 167.3, 167.2, 160.1, 158.0, 148.0, 131.8, 129.6, 120.4, 117.2, 112.8, 108.5, 107.4, 103.6, 102.0; TOF-HRMS *m/z* [M − H]^−^, calcd for C_15_H_11_O_5_: 271.0612; found: 271.0607.

#### 3.2.12. Preparation of 2′-Hydroxy-2,3′,4,5′-tetraisopropoxystilbene (**17**)

Compound **14** (121 mg, 0.27 mmol) and *p*-TsOH·H_2_O (15 mg, 0.09 mmol) were dissolved in methanol (1 mL). Then 30% hydrogen peroxide (0.07 mL, 0.54 mmol) was added at 0 °C, and the reaction was then warmed to room temperature and stirred for 1 h. Half-saturated sodium sulfite solution (1 mL) was added, and the mixture was extracted with CH_2_Cl_2_ (3 × 5 mL). The organic phase was washed with brine, dried over Na_2_SO_4_, filtered and concentrated under reduced pressure to give a crude product. Purification with preparative TLC (10% EtOAc/hexanes) gave **17** (78 mg, 66%). Pale yellow solid; IR (UATR, cm^−1^): 3536, 2975, 1600; ^1^H-NMR (300 MHz, CDCl_3_), δ (ppm): 7.53 (d, *J* = 8.4 Hz, 1H), 7.38 (d, *J* = 16.8 Hz, 1H), 7.32 (d, *J* = 16.5 Hz, 1H), 6.74 (d, *J* = 2.7 Hz, 1H), 6.48 (dd, *J* = 10.8, 2.4 Hz, 1H), 6.46 (br s, 1H), 6.40 (d, *J* = 2.4 Hz, 1H), 4.49 (m, 4H), 1.31–1.40 (m, 24H); ^13^C-NMR (75 MHz, CDCl_3_), δ (ppm): 158.4, 156.5, 150.7, 145.0, 138.8, 127.4, 124.6, 124.1, 121.1, 120.8, 107.5, 104.9, 103.3, 102.6, 71.8, 71.1, 71.0, 69.9, 22.2, 22.14, 22.13, 22.10; TOF-HRMS *m/z* [M + H]^+^, calcd for C_26_H_37_O_5_: 429.2635; found: 429.2643.

#### 3.2.13. Preparation of 3-(2,4-Dihydroxyphenyl)-6,8-dihydroxyisochroman-1-one (**18**)

To a solution of **15** (104 mg, 0.23 mmol) in dry CH_2_Cl_2_ (2 mL), a solution of BBr_3_ (0.96 mL, 0.96 mmol) in CH_2_Cl_2_ was added under argon at −78 °C, and the mixture was stirred for 20 min. After completion of the reaction, water (5 mL) was added, and the mixture was extracted with EtOAc (3 × 5 mL). The organic phase was washed with brine, dried over Na_2_SO_4_, filtered and concentrated under reduced pressure to give a crude product. Purification with column chromatography eluting with 5% MeOH/CH_2_Cl_2_ gave **18** (13 mg, 20%). Light brown solid, mp 210–212 °C; IR (UATR, cm^−1^): 3340, 3190, 1611; ^1^H-NMR (300 MHz, acetone-*d*_6_), δ (ppm): 7.28 (d, *J* = 8.4 Hz, 1H), 6.48 (d, *J* = 2.1 Hz, 1H), 6.43 (dd, *J* = 8.4, 2.4 Hz, 1H), 6.35 (br s, 1H), 6.30 (d, *J* = 2.1 Hz, 1H), 5.83 (dd, *J* = 12, 3 Hz, 1H), 3.29 (dd, *J* = 16.5, 12.3 Hz, 1H), 3.04 (dd, *J* = 16.5, 3.3 Hz, 1H); ^13^C-NMR (75 MHz, acetone-*d*_6_), δ (ppm): 171.1, 165.13, 165.07, 159.5, 156.3, 143.8, 128.9, 117.2, 107.9, 107.3, 103.4, 101.95, 101.88, 76.5, 34.4; TOF-HRMS *m/z* [M + Na]^+^, calcd for C_15_H_12_NaO_6_: 311.0526; found: 311.0529.

#### 3.2.14. Preparation of 2′-(*E*)-Carbethoxyethenyl-2,3′,4,5′-tetraisopropoxystilbene (**19**)

A solution of **14** in dry CH_2_Cl_2_ (4 mL) was added to EtO_2_CCH=PPh_3_ (176 mg, 0.51mmol) at 0 °C under argon. Then the reaction mixture was allowed to warm to room temperature and stirred overnight. After completion of the reaction, water (5 mL) was added, and the mixture was extracted with EtOAc (3 × 5 mL). The organic layer was washed with brine, dried over Na_2_SO_4_, filtered and concentrated under reduced pressure to give a crude product. Purification with column chromatography eluting with 50% CH_2_Cl_2_/hexanes gave **19** (134 mg, 68%). Pale yellow oil; IR (UATR, cm^−1^): 2977, 2932, 1706, 1589; ^1^H-NMR (300 MHz, CDCl_3_), δ (ppm): 8.03 (d, *J* = 16.2 Hz, 1H), 7.45 (d, *J* = 8.4 Hz, 1H), 7.37 (d, *J* = 16.2 Hz, 1H), 7.15 (d, *J* = 16.2 Hz, 1H), 6.70 (d, *J* = 2.1 Hz, 1H), 6.50 (d, *J* = 16.2 Hz, 1H), 6.49 (dd, *J* = 8.4, 2.4 Hz, 1H), 6.48 (br s, 1H), 6.39 (d, *J* = 2.1 Hz, 1H), 4.58 (m, 4H), 4.24 (q, *J* = 7.1 Hz, 2H), 1.28–1.40 (m, 27H); ^13^C-NMR (75 MHz, CDCl_3_), δ (ppm): 168.2, 159.4, 158.7, 156.7, 142.3, 139.0, 128.4, 127.4, 125.6, 120.3, 120.1, 115.8, 107.2, 105.6, 102.8, 100.8, 71.0, 70.7, 69.9, 69.8, 59.9, 22.09, 22.04, 22.00, 14.3; TOF-HRMS *m/z* [M + H]^+^, calcd for C_31_H_43_O_6_: 511.3054; found: 511.3077.

#### 3.2.15. Preparation of 3′,5′-Diacetoxy-2,4-diisopropoxystilbene (**20**)

Et_3_N (0.05 mL, 0.33 mmol) and acetic anhydride (0.03 mL, 0.33 mmol) were added to a solution of **5** (50 mg, 0.15 mmol) in CH_2_Cl_2_ (2 mL) at room temperature. The reaction mixture was stirred for 2 h. Water (5 mL) was added, and the reaction mixture was extracted with EtOAc (3 × 5 mL). The organic phase was washed with brine, dried over Na_2_SO_4_, filtered and concentrated under reduced pressure to give a crude product. Purification with column chromatography eluting with 20% EtOAc/hexanes gave **20** (46 mg, 74%). White solid, mp 65–67 °C; IR (UATR, cm^−1^): 2978, 1768, 1599; ^1^H-NMR (300 MHz, CDCl_3_), δ (ppm): 7.41 (d, *J* = 8.4 Hz, 1H), 7.34 (d, *J* = 16.5 Hz, 1H), 7.08 (d, *J* = 2.1 Hz, 2H), 6.94 (d, *J* = 16.2 Hz, 1H), 6.78 (t, *J* = 2.1 Hz, 1H), 6.46 (dd, *J* = 8.1, 2.4 Hz, 1H), 6.45 (br s, 1H), 4.51 (m, 2H), 2.25 (s, 6H), 1.36 (d, *J* = 6 Hz, 6H), 1.31 (d, *J* = 6 Hz, 6H); ^13^C-NMR (75 MHz, CDCl_3_), δ (ppm): 168.7, 158.8, 156.6, 151.0, 140.7, 127.6, 125.8, 124.6, 119.4, 116.3, 113.3, 106.9, 102.5, 70.6, 69.7, 21.9, 21.8, 20.8; TOF-HRMS *m/z* [M + Na]^+^, calcd for C_24_H_28_NaO_6_: 435.1778; found: 435.1796.

#### 3.2.16. Preparation of 3′,5′-Diacetoxy-5-formyl-2,4-diisopropoxystilbene (**21**)

POCl_3_ (0.16 mL, 1.77 mmol) was stirred with dry DMF (1 mL) at room temperature for 2 h under argon. The solution of **20** (73 mg, 0.18 mmol) in dry DMF (1 mL) was added at 0 °C. Then the reaction mixture was allowed to warm to room temperature and stirred overnight. After completion of the reaction, cool water (5 mL) was added, and the reaction mixture was extracted with EtOAc (3 × 5 mL). The organic layer was washed with water (7 × 5 mL) and brine, dried over Na_2_SO_4_, filtered and concentrated under reduced pressure to give a crude product, which was purified by preparative TLC (20% EtOAc/hexanes) to furnish **21** (46 mg, 60%). Viscous yellow oil; IR (UATR, cm^−1^): 2979, 1768, 1671, 1593; ^1^H-NMR (300 MHz, CDCl_3_), δ (ppm): 10.32 (s, 1H), 8.03 (s, 1H), 7.27 (d, *J* = 16.5 Hz, 1H), 7.08 (d, *J* = 1.8 Hz, 2H), 7.03 (d, *J* = 16.5 Hz, 1H), 6.81 (t, *J* = 2.1 Hz, 1H), 6.44 (s, 1H), 4.66 (m, 2H), 2.29 (s, 6H), 1.43 (d, *J* = 6 Hz, 6H), 1.40 (d, *J* = 6 Hz, 6H); ^13^C-NMR (75 MHz, CDCl_3_), δ (ppm): 188.1, 168.7, 161.8, 161.4, 151.0, 140.1, 126.6, 126.3, 124.2, 119.6, 118.8, 116.4, 113.8, 98.1, 71.2, 70.9, 21.67, 21.66, 20.8; TOF-HRMS *m/z* [M + Na]^+^, calcd for C_25_H_28_NaO_7_: 463.1727; found: 463.1743.

#### 3.2.17. Preparation of 3′,5′-Diacetoxy-5-carboxy-2,4-diisopropoxystilbene (**22**)

A solution of NaClO_2_ (85 mg, 0.93 mmol) and NaH_2_PO_4_·2H_2_O (145 mg, 0.93 mmol) in water (0.5 mL) was added to a solution of **21** (51 mg, 0.12 mmol) and 2-methyl-2-butene (0.04 mL, 0.49 mmol) in acetone (0.5 mL) at room temperature. The reaction mixture was stirred for 1 h. After completion, water (5 mL) was added, and the reaction mixture was extracted with EtOAc (3 × 5 mL). The organic layer was washed with brine, dried over Na_2_SO_4_, filtered and concentrated under reduced pressure to give a crude product, which was purified by column chromatography on silica (40% EtOAc/hexanes) to furnish **22** (39 mg, 73%). White solid, mp 106–108 °C; IR (UATR, cm^−1^): 3267, 2980, 1769, 1732, 1601; ^1^H-NMR (300 MHz, CDCl_3_), δ (ppm): 8.35 (s, 1H), 7.26 (d, *J* = 16.5 Hz, 1H), 7.09 (d, *J* = 2.1 Hz, 2H), 7.08 (d, *J* = 16.5 Hz, 1H), 6.82 (t, *J* = 2.1 Hz, 1H), 6.50 (s, 1H), 4.83 (sept, *J* = 6 Hz, 1H), 4.65 (sept, *J* = 6 Hz, 1H), 2.31 (s, 6H), 1.51 (d, *J* = 6 Hz, 6H), 1.45 (d, *J* = 6 Hz, 6H); ^13^C-NMR (75 MHz, CDCl_3_), δ (ppm): 168.9, 165.3, 160.2, 157.2, 151.2, 140.1, 132.1, 127.5, 123.9, 121.4, 116.8, 114.2, 110.9, 99.0, 74.2, 71.4, 21.92, 21.88, 21.0; TOF-HRMS *m/z* [M + Na]^+^, calcd for C_25_H_28_NaO_8_: 479.1676; found: 479.1695.

#### 3.2.18. Preparation of 5-Formyl-2,3′,4,5′-tetrahydroxystilbene (**23**)

A solution of BCl_3_ (0.42 mL, 0.42 mmol) was added to a solution of **21** (30 mg, 0.07 mmol) in CH_2_Cl_2_ (2 mL) at −78 °C under argon. Then it was allowed to warm to room temperature and stirred overnight. Water (5 mL) was then added, and the reaction mixture was extracted with EtOAc (3 × 5 mL). The EtOAc was washed with brine, dried over Na_2_SO_4_, filtered and concentrated under reduced pressure to give a crude product which was purified by preparative TLC (40% EtOAc/hexanes) to give **23** (15 mg, 78%). Yellow solid, decomposed >205 °C; IR (UATR, cm^−1^): 3208, 1626; ^1^H-NMR (300 MHz, methanol-*d*_4_), δ (ppm): 9.69 (s, 1H), 7.76 (s, 1H), 7.27 (d, *J* = 16.5 Hz, 1H), 6.95 (d, *J* = 16.5 Hz, 1H), 6.47 (d, *J* = 2.4 Hz, 2H), 6.25 (s, 1H), 6.16 (t, *J* = 2.1 Hz, 1H); ^13^C-NMR (75 MHz, methanol-*d*_4_), δ (ppm): 194.9, 167.8, 164.8, 159.6, 141.6, 133.2, 128.4, 123.8, 121.0, 115.5, 105.9, 103.7, 102.7; TOF-HRMS *m/z* [M − H]^−^, calcd for C_15_H_11_O_5_: 271.0612; found: 271.0603.

#### 3.2.19. Preparation of 5-Carboxy-2,3′,4,5′-tetrahydroxystilbene (**24**)

A solution of BCl_3_ (0.88 mL, 0.88 mmol) was added to a solution of **22** (68 mg, 0.15 mmol) in CH_2_Cl_2_ (2 mL) at −78 °C under argon. Then the reaction was allowed to warm to room temperature and stirred overnight. Water (5 mL) was added, and the mixture was extracted with EtOAc (3 × 5 mL). The organic layer was washed with brine, dried over Na_2_SO_4_, filtered and concentrated under reduced pressure to give a crude product which was purified by Sephadex LH20 (methanol) to give **24** (27 mg, 63%). Yellow solid, decomposed >230 °C; IR (UATR, cm^−1^): 3337, 1616; ^1^H-NMR (300 MHz, methanol-*d*_4_), δ (ppm): 8.01 (s, 1H), 7.24 (d, *J* = 16.5 Hz, 1H), 6.92 (d, *J* = 16.5 Hz, 1H), 6.49 (d, *J* = 2.1 Hz, 2H), 6.36 (s, 1H), 6.19 (t, *J* = 2.1 Hz, 1H); ^13^C-NMR (75 MHz, methanol-*d*_4_), δ (ppm): 173.5, 164.2, 162.8, 159.6, 141.6, 130.0, 128.5, 123.8, 118.9, 106.3, 105.8, 103.3, 102.7; TOF-HRMS *m/z* [M − H]^−^, calcd for C_15_H_11_O_6_: 287.0561; found: 287.0549.

#### 3.2.20. Preparation of 5-Formyl-3′,5′-dihydroxy-2,4-diisopropoxystilbene (**25**)

To **21** (59 mg, 0.12 mmol), KOH (5% in EtOH, 1 mL) was added at room temperature, and the reaction mixture was stirred for 10 min. After completion of the reaction, the reaction was extracted with EtOAc (3 × 5 mL). The EtOAc phase was washed with brine, dried over Na_2_SO_4_, filtered and concentrated under reduced pressure to give a crude product. Purification with column chromatography eluting with 50% EtOAc in hexanes gave **25** (41 mg, 93%). Yellow solid, mp 186–188 °C; IR (UATR, cm^−1^): 3355, 2979, 1588; ^1^H-NMR (300 MHz, acetone-*d*_6_), δ (ppm): 10.32 (s, 1H), 7.99 (s, 1H), 7.30 (d, *J* = 16.5 Hz, 1H), 7.05 (d, *J* = 16.5 Hz, 1H), 6.80 (s, 1H), 6.59 (d, *J* = 2 Hz, 2H), 6.29 (t, *J* = 2 Hz, 1H), 4.91 (m, 2H), 1.43 (d, *J* = 6.3 Hz, 6H), 1.41 (d, *J* = 6.4 Hz, 6H); ^13^C-NMR (75 MHz, acetone-*d*_6_), δ (ppm): 188.0, 162.7, 162.3, 159.6, 140.9, 129.2, 126.6, 122.9, 121.1, 120.1, 105.8, 102.9, 100.0, 72.1, 71.8, 22.2, 22.1; TOF-HRMS *m/z* [M + H]^+^, calcd for C_21_H_25_O_5_: 357.1696; found: 357.1712.

#### 3.2.21. Preparation of 5-Carboxy-3′,5′-dihydroxy-2,4-diisopropoxystilbene (**26**)

To **22** (30 mg, 0.07 mmol), KOH (5% in EtOH, 1 mL) was added at room temperature, and the reaction mixture was stirred for 10 min. The reaction was acidified with 2 N HCl to pH 5. The material was extracted with EtOAc (3 × 5 mL), and the combined organic layers were dried over Na_2_SO_4_, filtered, and concentrated under reduced pressure to provide **26** (10 mg, 40%). Light brown solid, mp 112–114 °C; ^1^H-NMR (300 MHz, acetone-*d*_6_), δ (ppm): 8.23 (s, 1H), 7.31 (d, *J* = 16 Hz, 1H), 7.07 (d, *J* = 16 Hz, 1H), 6.88 (s, 1H), 6.59 (d, *J* = 2 Hz, 2H), 6.30 (t, *J* = 2 Hz, 1H), 5.04 (sept, *J* = 6 Hz, 1H), 4.89 (sept, *J* = 6 Hz, 1H), 1.46 (d, *J* = 6.3 Hz, 6H), 1.43 (d, *J* = 6 Hz, 6H); ^13^C-NMR (75 MHz, acetone-*d*_6_), δ (ppm): 165.7, 160.8, 159.6, 158.5, 140.8, 131.5, 129.6, 122.9, 122.0, 112.7, 108.0, 103.0, 101.1, 74.4, 71.9, 22.2, 22.1; TOF-HRMS *m/z* [M − H]^−^, calcd for C_21_H_23_O_6_: 357.1500; found: 371.1502.

#### 3.2.22. Preparation of 3′,5′-Diacetoxy-5-hydroxy-2,4-diisopropoxystilbene (**27**)

A solution of 30% hydrogen peroxide (0.03 mL, 0.192 mmol) was added to a methanolic mixture of **21** (44 mg, 0.10 mmol) and *p*-TsOH·H_2_O (5 mg, 0.03 mmol) at 0 °C. The reaction mixture was allowed to warm to room temperature and stirred for 1 h. Half-saturated sodium sulfite solution (1 mL) was added, and the mixture was extracted with CH_2_Cl_2_ (3 × 5 mL). The CH_2_Cl_2_ layer was washed with brine, dried over Na_2_SO_4_, filtered and concentrated under reduced pressure to give a crude product. Purification with preparative TLC (30% EtOAc/hexanes) gave **27** (19 mg, 47%). Pale yellow solid, mp 138–140 °C; IR (UATR, cm^−1^): 3525, 2977, 1767; ^1^H-NMR (300 MHz, CDCl_3_), δ (ppm): 7.34 (d, *J* = 16.2 Hz, 1H), 7.13 (s, 1H), 7.07 (d, *J* = 2.1 Hz, 2H), 6.87 (d, *J* = 16.2 Hz, 1H), 6.79 (t, *J* = 1.8 Hz, 1H), 6.50 (s, 1H), 4.54 (sept, *J* = 6 Hz, 1H), 4.33 (sept, *J* = 6 Hz, 1H), 2.30 (s, 6H), 1.36 (d, *J* = 6.3 Hz, 6H), 1.34 (d, *J* = 6.3 Hz, 6H); ^13^C-NMR (75 MHz, CDCl_3_), δ (ppm): 169.0, 151.1, 149.4, 145.0, 141.4, 140.6, 125.3, 125.2, 120.9, 116.5, 113.7, 111.3, 103.7, 73.3, 71.8, 22.2, 22.0, 21.0; TOF-HRMS *m/z* [M + Na]^+^, calcd for C_24_H_28_NaO_7_: 451.1727; found: 451.1723.

### 3.3. Biological Activities

In this study, statistical analysis of the biological data was performed using the Student's *t*-test. All results were expressed as arithmetic mean ± standard deviation (SD).

#### 3.3.1. DPPH Radical Assay

The experiment was performed according to an established method [33]. The experiment was performed in a 96-well plate. The reduction of the DPPH was measured by reading the absorbance at 510 nm with a Perkin Elmer Victor^3^ multilabel counter. Oxyresveratrol and Trolox were used as positive controls.

#### 3.3.2. Superoxide Radical Assay

The assay was based on the capacity of the sample to inhibit the reduction of nitroblue tetrazolium (NBT) in the riboflavin-light-NBT system [18,34]. Oxyresveratrol and Trolox were used as positive controls.

#### 3.3.3. Inhibitory Effect on Supercoiled DNA Breakage

The assay was performed as previously described [18]. This assay measured the ability of the test sample to protect DNA against damage induced by the photochemical reaction of riboflavin, with oxyresveratrol and Trolox used as positive controls.

#### 3.3.4. Determination of Anti-Herpes Simplex Virus Activity

Anti-HSV activities of the compounds were determined using a modified plaque reduction assay, with acyclovir as a positive control [35].

#### 3.3.5. Neuraminidase (NA) Inhibition Assay

This assay was performed using the method previously described by Potier and co-workers [36]. The fluorescence was measured using SpectraMax M5 multi-detection microplate reader (Molecular Devices, Sunnyvale, CA, USA) with excitation and emission wave lengths of 365 and 450 nm, respectively. Oseltamivir was used as a positive control.

#### 3.3.6. Determination of α-Glucosidase Inhibitory Activity

The assay was performed in a 96-well plate and based on the capacity of the sample to inhibit the hydrolysis of *p*-nitrophenyl-α-d-glucoside (PNPG) by α-glucosidase to release *p*-nitrophenol (PNP), a yellow color agent that can be monitored at 405 nm [20]. Acarbose was used as a positive control.

#### 3.3.7. Determination of Cytotoxic Activity

The assays for cytotoxicity were carried out according to a previous report [37]. Cell viability was determined using MTT assay. Doxorubicin was used as the positive control.

## 4. Conclusions

A total of 26 derivatives were prepared from oxyresveratrol (**1**) through several types of reactions. Selective di-*O*-alkylation is possible because the two hydroxy groups at C-2 and C-4 of ring A of **1** are obviously more reactive than those on ring B. Electrophilic substitution on the B ring is more facile than on the A ring. However, the relative reactivity can be reversed by manipulating the type and the number of protecting groups. Through this strategy, electrophilic substitution reactions can be selectively directed to ring A or B. This finding has provided a new and useful approach for the future chemical modification of similarly oxygenated aromatic structures.

Compound **1** and analogs **2**–**27** were evaluated for a number of biological activities, including free radical scavenging activity, DNA protective properties, antiherpetic activity, inhibition of α-glucosidase and neuraminidase, and cytotoxicity against some cancer cell lines. Some of the derivatives were equally active or more potent than the parent compound. It is interesting to note that the partially etherified products 3′,5′-dihydroxy-2,4-dimethoxystilbene **(2**) and 5′-hydroxy-2,3′,4-trimethoxystilbene (**3**) demonstrated encouraging antiherpetic and DNA protective activities. Compared with the parent compound, **2** and **3** should have higher lipophilicity, and thus should have greater potential as preventive agents for neurodegenerative diseases. 5′-Hydroxy-2,3′,4,-triisopropoxystilbene (**6**), an etherified analog with retained anti-α-glucosidase activity but with a potentially slower rate of metabolism than that of **1**, should be a better lead for oral anti-diabetic drug development. In addition, the mixed esterified/etherified derivative 3′,5′-diacetoxy-2,4-diisopropoxystilbene (**20**) displayed pronounced *in vitro* cytotoxicity against HeLa cells, and warrants further investigation in animals.

## Figures and Tables

**Table 1:** IC_50_ values (μM) for free radical scavenging activities and DNA protective properties of **1** and derivatives.

Compound	Free Radical Scavenging Activity		DNA Protective Activity
	DPPH	Superoxide	
**1**	11.7 ± 0.4	303.1 ± 7.9	43.3 ± 6.7
**2**	77.0 ± 6.4 *	nd #	39.4 ± 1.2
**3**	nd	120.1 ± 14.9 *	6.3 ± 0.8 *
**5**	147.7± 9.8 *	nd	59.1 ± 9.9
**10**	9.7 ± 0.2 *	154.9 ± 14.7 *	19.9 ± 2.8 *
**11**	19.4 ± 3.1 *	81.9 ± 12.3 *	28.6 ± 3.8 *
**12**	14.7 ± 0.6 *	98.4 ± 7.0 *	32.0 ± 1.4 *
**16**	16.5 ± 2.8 *	43.4 ± 4.4 *	32.2 ± 4.8
**17**	11.7 ± 0.3	nd	nd
**18**	nd	107.3 ± 8.7 *	28.3 ± 4.3 *
**22**	nd	17.7 ± 3.5 *	81.4 ± 4.2 *
**23**	nd	88.3 ± 9.7 *	54.7 ± 8.3
**24**	48.3 ± 5.8 *	38.6 ± 1.4 *	79.4 ± 14.9 *
**25**	nd	nd	111.8 ± 7.3 *
**26**	nd	nd	104.7 ± 8.5 *
**27**	7.0 ± 0.2 *	nd	18.6 ± 3.7 *
**Trolox**	8.7 ± 1.1 *	293.5 ± 19.3	113.1 ± 4.6 *

#: nd = not determined due to <80% inhibition at 100 μg/mL.; *: Significantly different from that of **1** (*p* < 0.05).

**Table 2:** Anti-herpetic activity of **1** and derivatives.

Compounds	IC_50_ (µM)
**1**	147.1 ± 16.8
**2**	40.2 ± 6.8 *
**3**	32.8 ± 5.9 *
**6**	107.0 ± 9.8 *
**10**	182.3 ± 28.9
**14**	130.3 ± 14.9
**15**	123.5 ± 19.3
**18**	289.3 ± 40.8 *
**21**	188.3 ± 25.1
**22**	207.4 ± 16.9 *
**23**	344.7 ± 32.5 *
**26**	226.8 ± 35.7 *
ACV	1.6 ± 0.0 *

* Significantly different from that of **1** (*p* < 0.05).

**Table 3:** Cytotoxicity against cancer cells of **1** and some derivatives.

Compounds	IC_50_ (μM)			
	T47-D	HeLa	A549	H69AR
**1**	152.7 ± 4.5	126.2 ± 0.3	159.8 ± 0.0	nd #
**2**	114.2 ± 2.0 *	28.4 ± 7.0 *	117.0 ± 11.1 *	180.7 ± 3.4
**3**	87.1 ± 9.5 *	13.1 ± 2.1 *	52.4 ± 0.9 *	36.7 ± 3.3
**4**	nd	12.1 ± 1.5 *	nd	26.8 ± 7.6
**5**	117.4 ± 11.9 *	11.0 ± 2.7 *	55.5 ± 3.5 *	39.2 ± 6.5
**6**	37.7 ± 12.9 *	25.7 ± 2.1 *	28.8 ± 5.1 *	54.5 ±3.0
**8**	103.0 ± 4.3 *	nd	nd	nd
**10**	88.6 ± 2.9 *	100.9 ± 4.7 *	137.7 ± 8.4 *	nd
**12**	156.6 ± 6.0	33.4 ± 2.9 *	120.7 ± 2.9 *	111.2 ± 3.1
**13**	nd	18.7 ± 4.2 *	nd	65.7 ± 3.2
**14**	nd	69.1 ± 3.3 *	nd	nd
**15**	101.4 ± 9.1 *	83.2 ± 9.1 *	nd	nd
**16**	99.3 ± 3.1 *	74.4 ± 4.8 *	138.0 ± 3.1 *	nd
**20**	81.3 ± 0.6 *	5.7 ± 0.6 *	51.7 ± 1.7 *	38.0 ± 2.2
**21**	54.5 ± 2.0 *	18.9 ± 0.7 *	71.3 ± 3.2 *	nd
**23**	82.9 ± 2.2 *	77.9 ± 2.1 *	143.2 ± 3.3 *	nd
**25**	44.7 ± 2.6 *	22.4 ± 0.4 *	75.1 ± 0.7 *	111.0 ± 2.3
**27**	83.2 ± 10.6 *	79.8 ± 4.7 *	104.4 ± 5.3 *	nd
**Doxorubicin**	0.5 ± 0.00 *	0.7 ± 0.2 *	0.4 ± 0.1 *	21.7 ± 0.9

#: nd = not determined due to <50 % inhibition at 50 μg/mL.; *: Significantly different from that of **1** (*p* < 0.05). Cell lines: T47D = human hormone-dependent breast cancer, HeLa = human cervical adenocarcinoma, A549 = human non-small-cell lung carcinoma, H69AR = human multidrug-resistant small-cell lung carcinoma.
